# Influenza-induced Tpl2 expression within alveolar epithelial cells is dispensable for host viral control and anti-viral immunity

**DOI:** 10.1371/journal.pone.0262832

**Published:** 2022-01-20

**Authors:** Kara D. Wyatt, Demba Sarr, Kaori Sakamoto, Wendy T. Watford

**Affiliations:** 1 Department of Infectious Diseases, University of Georgia, Athens, Georgia, United States of America; 2 Department of Pathology, University of Georgia, Athens, Georgia, United States of America; Washington State University, UNITED STATES

## Abstract

Tumor progression locus 2 (Tpl2) is a serine/threonine kinase that regulates the expression of inflammatory mediators in response to Toll-like receptors (TLR) and cytokine receptors. Global ablation of Tpl2 leads to severe disease in response to influenza A virus (IAV) infection, characterized by respiratory distress, and studies in bone marrow chimeric mice implicated Tpl2 in non-hematopoietic cells. Lung epithelial cells are primary targets and replicative niches of influenza viruses; however, the specific regulation of antiviral responses by Tpl2 within lung epithelial cells has not been investigated. Herein, we show that Tpl2 is basally expressed in primary airway epithelial cells and that its expression increases in both type I and type II airway epithelial cells (AECI and AECII) in response to influenza infection. We used *Nkx2*.*1*-cre to drive Tpl2 deletion within pulmonary epithelial cells to delineate epithelial cell-specific functions of Tpl2 during influenza infection in mice. Although modest increases in morbidity and mortality were attributed to cre-dependent deletion in lung epithelial cells, no alterations in host cytokine production or lung pathology were observed. *In vitro*, Tpl2 inhibition within the type I airway epithelial cell line, LET1, as well as genetic ablation in primary airway epithelial cells did not alter cytokine production. Overall, these findings establish that Tpl2-dependent defects in cells other than AECs are primarily responsible for the morbidity and mortality seen in influenza-infected mice with global Tpl2 ablation.

## Introduction

Influenza is one of the most common causes of respiratory infections [1]. Within the United States, approximately 200,000 hospitalizations occur during a typical flu season [1]. Seasonal influenza poses a substantial threat to global health, given the current global coronavirus pandemic, and the at-risk population may be even greater, including those with co-morbidities, like COVID-19, who are not typically at risk for influenza complications [2].

Interferons (IFNs) are host restriction factors that ‘interfere’ with viral replication [3]. There are three major subsets of IFNs, including type I (IFN-α/IFN-β), type II (IFN-γ), and type III (IFN-λs). It is well-established that influenza infections produce a robust type I (IFN-α/IFN-β) IFN response [4]. However, type III IFNs are a genetically distinct subset of IFNs, and type III IFN receptor expression is restricted primarily to epithelial cells. Type III IFNs are now appreciated to be the predominant IFN produced during influenza A infection [5], and they play an essential, non-redundant role in protecting these primary targets of influenza [6–8]. Specifically, IFN-λ is critical for controlling early viral replication without driving immune activation and tissue damage. Therefore, regulation of IFNs is crucial to maintaining an appropriate balance between viral control and host-induced pathology.

One known regulator of IFNs is the serine-threonine kinase, Tumor progression locus 2 (Tpl2), also known as *Cot* or MAP3K8. Tpl2 is broadly expressed in hematopoietic and non-hematopoietic cells [9]. Tpl2 transduces intracellular signaling through ERK, JNK, p38, and the PI3K-AKT-mTOR axis [9–16], and it regulates proliferation and gene expression in a context-dependent manner [9]. Tpl2 negatively regulates IFN-β production in macrophages but positively regulates IFN-α and IFN-β production in plasmacytoid dendritic cells (pDCs) in response to TLR stimulation [17]. Tpl2 also promotes the induction of IFN-λ in pDCs [18]. Previous studies have established an important role for Tpl2 in promoting inflammatory responses including the production of TNF [19, 20], IFN-γ [19, 20], and IL-1β [21].

Kuriakose *et al*. demonstrated that *Tpl2*^*-/-*^ mice have enhanced susceptibility to a low pathogenic strain of influenza A (x31; H3N2), and the increased susceptibility was caused by Tpl2 deficiency within radioresistant cells [18]. Multiple lines of evidence now support that Tpl2 plays critical regulatory roles within radioresistant non-hematopoietic cells [22, 23]. For instance, Tpl2 signaling within intestinal subepithelial myofibroblasts modulates hepatocyte growth factor and epithelial cell proliferation during colitis-induced colorectal carcinogenesis [23]. Despite the designation as a viral restriction factor *in vitro* and *in vivo* [18, 24], the role of Tpl2 within lung epithelial cells during a viral infection has not been investigated.

Pulmonary alveoli are known replicative niches of influenza [25] and are comprised of two major cell types: type I and type II alveolar epithelial cells (AECI/AECII). AECI are fused with capillary endothelial cells to form the alveolo-capillary barrier structure for gas exchange [26]. AECI are also key contributors to pulmonary fibrosis, which can lead to fatal deficiencies in lung function [26]. AECII are cuboidal cells that are crucial for surfactant production and lung homeostasis [27].

Tpl2 protects against influenza infection *in vivo* [18]; however, there is limited information about the cellular mechanism responsible. In this study, we generated a lung-specific conditional *Tpl2*-knockout mouse model to study the contribution of Tpl2 within respiratory epithelial cells using the *Nkx2*.*1* promoter to drive cre recombinase expression. In addition, we utilized the immortalized murine lung epithelial type I cell line (LET1) and primary AECIIs to model epithelial cell responses to influenza A virus. We demonstrate that cre-dependent deletion of *Tpl2* expression within epithelial cells only modestly enhances mortality after influenza infection, without substantially altering virus titers or inflammatory cytokine production. *In vitro* analysis of an AECI cell line and primary lung epithelial cells showed that *Tpl2* is dispensable for influenza-induced interferon responses. Overall, this study suggests that *Tpl2* ablation in radioresistant cells other than pulmonary epithelial cells is responsible for the poor clinical outcome in mice with global Tpl2 ablation.

## Materials and methods

### Mice and viruses

Wild-type (WT) C57BL/6J mice were purchased from The Jackson Laboratory. *Tpl2*-deficient mice were backcrossed to C57BL6/J for more than ten generations and kindly provided by Dr. Philip Tsichlis and Thomas Jefferson University [10]. *Tpl2*^flox/flox^ mice were offered by Dr. George Kollias [23] and purchased from EMMA (EM:07150). Conditional ablation of *Tpl2* was limited to cells within the lung epithelium (AECI, AECII, club cells, bronchial epithelial cells, basal cells, ciliated epithelial cells, and goblet cells) and were generated by crossing *Tpl2*^flox/flox^ mice with *Nkx2*.*1*cre, respectively.

To enhance deletion efficiency of *Tpl2*, the resulting *Nkx2*.*1*cre^+/-^*Tpl2*^flox/flox^ and *Tpl2*^flox/flox^ mice were further crossed with *Tpl2*^*-/-*^ mice to generate experimental (cre+) and littermate control (cre) *Nkx2*.*1*cre^+^*Tpl2*^flox/-^ mice. Mice were housed in specific pathogen-free conditions in microisolator cages in the Coverdell Rodent Vivarium within the University of Georgia. Animals were confirmed *Helicobacter*-negative and used between six to nine weeks of age. To prevent sex-biases, both male and female mice were used in all experiments. All experiments involving mice were performed according to the University of Georgia Guidelines for Laboratory Animals and were approved by the UGA Institutional Animal Care and Use Committee. Mouse-adapted influenza virus A/HK-x31 (H3N2) stocks were provided by Dr. Mark Tompkins (University of Georgia).

### *In vivo* influenza infection

Age-matched, six- to nine-week-old mice were sedated with 2.5% Avertin and intranasally infected with 50 μl of influenza A/x31 (10^4^ PFU) diluted in PBS. To determine lung viral titers, lungs were harvested on 1, 3, 5, and 7 days post infection (dpi), placed in 1 ml of PBS, and dissociated with a bead mill homogenizer (Qiagen). Virus titers were quantified by plaque assays (described below). To assess susceptibility to influenza infection, mice were infected with 10^4^ pfu of influenza A/x31 and observed over a 14-day period. Body weights were recorded daily, and mice exhibiting severe signs of disease or more than 30% weight loss were euthanized. For morbidity and mortality measurements, researchers were blinded to mouse genotype until the end of the experiment.

### Epithelial cell isolation

The isolation of lung epithelial cells was adapted from the protocol of Sinha and Lowell [28] as follows: mice were sacrificed by intraperitoneal injection of 2.5% avertin with secondary mode of sacrifice performed by cutting the renal artery. Cardiac perfusion was performed with sterile PBS followed by intratracheal instillation of ~1 mL dispase (50 units/mL) followed by 0.6 ml of 1% low-melt agarose. Lungs were harvested and individual lobes cut and incubated in dispase for 45 min at room temperature on a horizontal rocker. Single-cell suspensions were prepared by teasing apart the airways in Complete DMEM (DMEM, 10% FBS, 1X Penicillin-Streptomycin, 10 mM HEPES) with DNase (10 μg/mL) and incubated for 10 min at room temperature. Single-cell suspensions were serially strained through 70 μM and 40 μM strainers, and centrifuged at 300 x *g* for 10 min at 4°C.

Pellets were resuspended in Complete DMEM without DNase and stained with the following biotinylated antibodies at 1:100 dilution (anti-CD45, anti-CD31, anti-Ter119, and anti-integrin B4/CD104, Biolegend). Magnetic depletion of biotinylated samples was performed using a Mouse Streptavidin RapidSpheres Isolation Kit (StemCell Technologies). Enriched cells were washed with Complete DMEM at 300 x *g* and stained for 10 min at 4°C with CD16/32 (purified, ebioscience, 1:100), EpCAM (PE, ebioscience, 1:100), CD45.2 (FITC, ebioscience, 1:100), and Podoplanin (APC, ebioscience, 1:100). Cells were washed, stained, and strained through a 35 μm filter cap tube (Falcon) before sorting for singlet EpCAM^+^CD45.2^-^ (AECII) or EpCAM^+^CD45.2^-^Podoplanin (AECI) cells on a Beckman Coulter MoFlo Astrios EQ.

### Leukocyte isolation

Naïve splenic T cells were isolated by disaggregating spleens [29] and sorting for CD4 (CD45.2+/CD4+) or CD8 (CD45.2/CD8+) cells using a Beckman Coulter MoFlo Astrios EQ. Bone marrow-derived macrophages (BMDMs) were generated from WT age- and sex-matched mice [21, 30]. BMDMs were cultured (2 × 10^6^/ml) in DMEM low-glucose medium containing 10% FBS, 100 μg/mL penicillin-streptomycin, and 2 mM L-glutamine on sterile Petri dishes for 7 days at 37°C. Media was supplemented with 10 ng/ml of macrophage colony-stimulating factor (M-CSF) (PeproTech). Fresh medium equal to half of the initial volume containing 10 ng/ml M-CSF was added on day 5. On day 7, cells were washed, and adherent cells were incubated with cell dissociation buffer (Invitrogen) for 10 min at 37°C. Peritoneal exudate cells were isolated from WT mice intraperitoneally injected with 1 ml of 3% thioglycollate. Peritoneal lavage was collected 4 hr post injection. Cells were adherence purified overnight, washed, and lysed directly on the plate. Neutrophils were isolated from the bone marrow of WT mice and purified by negative selection using a neutrophil enrichment kit (Stem Cell). All harvested cells were lysed using TRK lysis buffer (E.Z.N.A. Total RNA kit, Omega Biotek).

### *In vitro* cell culture

Madin-Darby Canine Kidney cells (MDCKs) were cultured in DMEM with 10% FBS, 2 mM L-glutamine, and 100 μM Gentamicin, and plated at 0.5 x 10^6^ cells overnight prior to plaque assays. LET1s were graciously supplied by Dr. Paul Thomas (St. Jude Children’s Research Hospital) [31]. LET1s were cultured in DMEM with 10% FBS, 2 mM L-glutamine, and 100 μM Penicillin-Streptomycin. All cell media were filter-sterilized, and cells were cultured at 37°C and 5% CO_2_ until confluency. MDCKs and LET1s were passaged using TrypLE Express (Gibco).

### *In vitro* influenza infection

Influenza infection of LET1s was performed by seeding 2.5 x 10^5^ cells per well. Cells were infected with influenza A/x31 at a multiplicity of infection (MOI) of 0.5 or 1 for 24 hr. LET1s were pre-treated for 30 min with DMSO or Tpl2 inhibitor (5 μM; Calbiochem, catalog number: 616373). Supernatants and RNA lysates were collected at 24 hr.

### *In vitro* influenza plaque assay

MDCKs (0.5 x 10^6^/well) were plated overnight. Lung homogenates from *Nkx2*.*1*cre*Tpl2*^*flox/-*^ mice were diluted at 1:10 in infection media (1X MEM, 1X Penicillin-Streptomycin diluted in dH_2_O with a pH of 7.4) and applied to MDCKs. After a 1hr infection, 2.4% Avicel and 2x Overlay Media (2X MEM, 40 mM HEPES, 4 mM L-glutamine, 0.15% Sodium Bicarbonate, 2X Penicillin-Streptomycin diluted in dH_2_O with a pH of 7.4) were combined at a 1:1 ratio and added to the cultures to facilitate plaque formation. Three dpi, cells were washed with PBS and fixed with a 60:40 acetone/methanol and counterstained with crystal violet [32].

### Protein measurements

Lungs were harvested from *Nkx2*.*1*cre*Tpl2*^*flox/-*^ mice, placed in 1 ml PBS and dissociated with a bead mill homogenizer (Qiagen). Lung homogenates were centrifuged at 500 x *g* for 5 min. Cytokines were quantified using a custom Procartaplex Immunoassay (Thermo-Scientific), IFN-α and IFN-β Luminescent ELISA kits (Invivogen) and Mouse Inflammation Cytometric Bead Array (CBA, BD Bioscience). IFN-λ3 (IL-28B) was measured using a colorimetric ELISA (Invitrogen). Hydroxyproline assay kit (Sigma, MAK357) was utilized to determine collagen levels in *Nkx2*.*1*cre*Tpl2*^*flox/-*^ lungs infected with influenza A/x31 for 8 days prior to harvesting.

### mRNA measurements

RNA was isolated from cell lines or lungs using an E.Z.N.A. Total RNA kit (Omega Bio-Tek, Norcross, GA) and converted to cDNA by high capacity cDNA reverse transcription kit (Life Technologies). Relative expression levels of *c-fos*, *IFNα1*, *IFNβ1*, *IFNλ3*, *Ptges2*, *Reg3g*, and *TIMP1* were measured using probes from Applied Biosystems (Grand Island, NY) and a SensiFAST Probe Hi-ROX kit (Bioline, Taunton, MA). Samples were run on a StepOnePlus qPCR machine (Applied Biosystems), and results computed relative to WT uninfected and actin control using the ΔΔC_T_ method.

### Complete blood count with automated differential

*Tpl2*^*flox/-*^ mice and *Nkx2*.*1*cre*Tpl2*^*flox/-*^ mice were infected with influenza A/x31 for 8.5 days prior to harvesting. Blood (100–200 μl) was collected into EDTA micro-volume tubes (Fisher Scientific) by terminal cardiac puncture from mice freshly euthanized with CO_2_. A complete blood count with automated differential was analyzed by the Clinical Pathology Lab at the Veterinary Teaching Hospital at the University of Georgia on a Heska Elements HT5.

### Pathology scoring

Lungs from mice infected with influenza A/x31 for 8.5 days were harvested and fixed in 10% neutral-buffered formalin for 24 hr at room temperature. Formalin-fixed lungs were placed in cassettes, processed routinely for paraffin embedding and sectioning at 4-μm thickness, and mounted onto glass slides. Sections were stained using Periodic Acid Schiff (PAS) reaction, counterstained with hematoxylin and Masson’s trichrome stain, or stained with hematoxylin and eosin (H&E) only.

Histologic sections were evaluated in a blinded manner by a board-certified, veterinary pathologist (K.S.) and scored according to the following criteria for inflammation: (A) Percent of lung affected; (B) Alveolar score, Alveolar edema score, Pleuritis score;: 1 = focal, 2 = multifocal, 3 = multifocal to coalescing, 4 = most of lobule affected; (C) Bronchiolar score: 1 = focal, 2 = multifocal, 3 = most of the bronchioles in a lobule affected, 4 = lobule diffusely affected; (D) PMN score; 1 = neutrophils compose up to 25% of cells in alveoli, 2 = 25–49%, 3 = 50–74%, 4 = 75%+; (E) Perivascular cuffing (PVC) score: 1 = vessel cuffed by 1 leukocyte layer, 2 = 2–5 cells thick, 3 = 6–9 cells thick, 4 = 10+ cells thick; (F) Vasculitis score: 1 = infiltration of vessel wall by leukocytes, 2 = infiltration and separation of smooth muscle cells, 3 = infiltration and fibrinoid change; (G) Interstitial pneumonia (IP) score: 1 = alveolar septa infiltrated and thickened by 1 leukocyte layer, 2 = thickened by 2 leukocyte layers, 3 = 3 leukocyte layers, 4 = 4 leukocyte layers. PAS-Masson’s Trichrome-stained histological samples were characterized then scored according to the following criteria for fibrosis: (A) Distribution of the fibrosis: Perivascular, Peribronchial, or Alveolar; (B) Thickness relative to the wall of the blood vessel (PV), bronchioles (PB), or alveolar septa: 1 = 1x thickness, 2 = 2x thickness, 3 = 3x thickness, 4 = 4x thickness, 5 = 5x thickness.

#### Immunofluorescence staining and confocal microscopy

Four micron lung sections were mounted onto glass slides, and immunostaining of Tpl2 or influenza nucleoprotein (NP) plus Tpl2 were performed as previously described [33], with slight modifications. Briefly, paraffin-embedded influenza-infected and uninfected mouse lung tissue sections were deparaffinized and rehydrated with xylene and alcohol gradient. Antigen-retrieval was performed with 0.1 M sodium citrate in a Pascal Pressure Cooker (DakoCytomation) for 20 min at 95°C. Slides were cooled for 30 minutes at room temperature, washed with distilled water followed by 1X Tris Buffered Saline with 0.1% Tween (1X TBST). Sections were permeabilized in 1X TBS with 0.5% Triton X-100 for 5 min followed by blocking in 1% normal horse serum for at least 30 minutes at room temperature in a humidified chamber. Sections were then incubated in primary antibodies (rabbit anti-mouse Tpl2, clone M20, Santa Cruz, 1/200; and mouse anti-NP antibody, clone IC5-1B7, BEI Resources, 1/500) overnight followed by appropriate FITC and 594-conjugated secondary antibodies (Vector Laboratories). Slides were washed in 1X TBST and mounted VectaShield^®^ Plus antifade mounting medium with DAPI (H2000). Digital images were acquired on a Nikon A1R confocal microscope (Nikon Eclipse Ti-E inverted microscope, Melville, NY).

### Statistics

*P* values were derived by either paired or unpaired t-tests or two-way ANOVA with Tukey’s multiple comparisons test as indicated using PRISM software. Pathology scores were analyzed using Kruskal-Wallis Test with Dunn’s multiple comparisons test. Differences were considered statistically significant if *p* ≤ 0.05. Data represent means ± SEM. Kaplan-Meier analysis using PRISM software was performed to determine percent survival of *Nkx2*.*1*cre*Tpl2*^*flox/-*^ infected with influenza virus, and the *p* value was determined using a Mantel-Cox test.

## Results

### Tpl2 is expressed in pulmonary epithelial cells, and expression increases upon influenza infection

**Fig 1:**
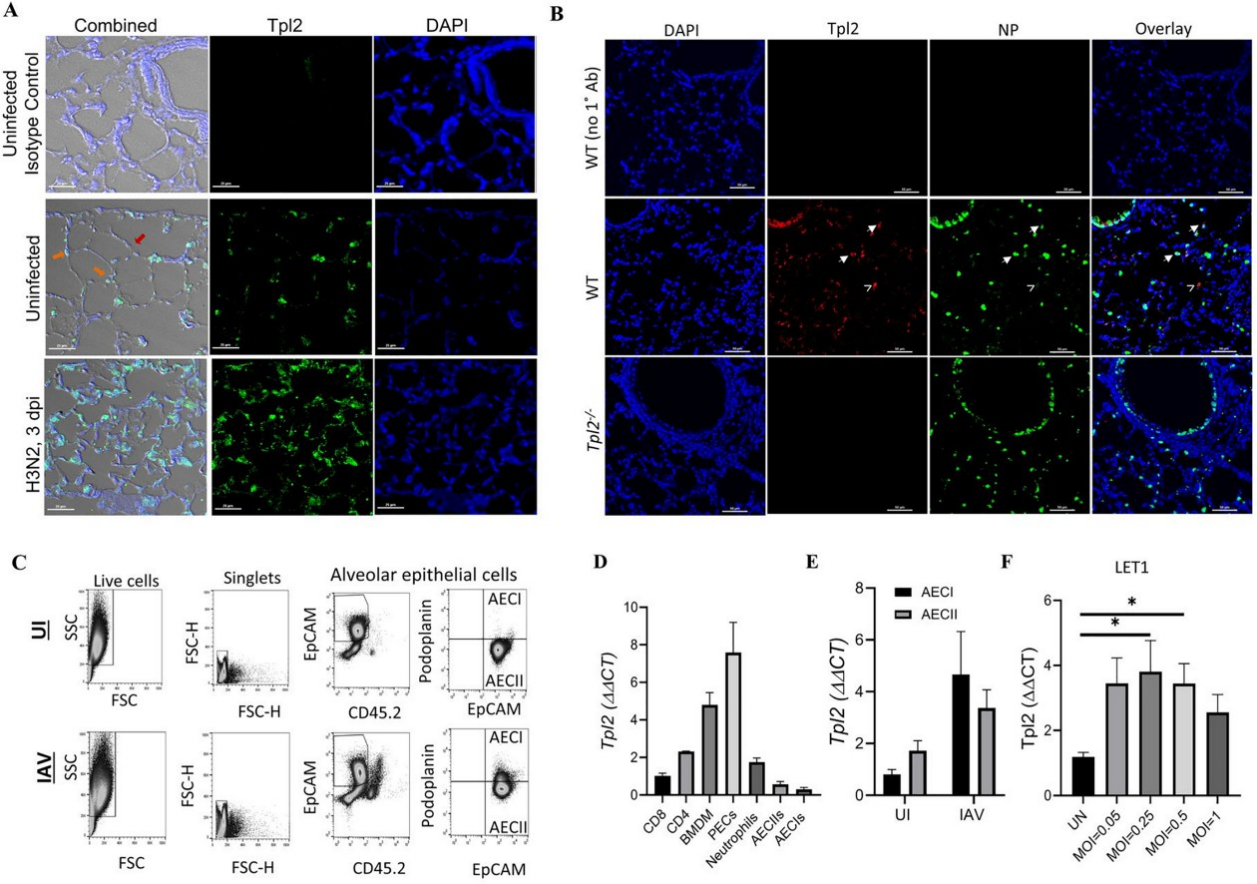
Tpl2 is expressed in pulmonary epithelial cells, and expression increases upon influenza infection. (A) Representative images of Tpl2 expression within 4-μm lung sections mounted onto glass slides from wild-type (WT) mice. WT mice were either uninfected or infected with 10^4^ PFU influenza A/ HK-x31 (x31) for 3 days. Lung sections were stained with rabbit anti-mouse IgG Isotype control or anti-mouse Tpl2 (Santa Cruz Biotechnology, clone M20) followed by goat anti-rabbit IgG AlexaFluor 488, and the nuclei were counterstained with DAPI. Slides were imaged using confocal microscopy. Data are representative of 3 experiments. Orange arrows—AECIIs, Red arrow—AECIs. (B) Representative images of influenza A/x31 nucleoprotein (NP) and Tpl2 expression in lung sections from WT and *Tpl2*^*-/-*^ mice infected with influenza for 3 days as in A. Cells were stained with anti-NP antibody (with AlexaFluor 488 secondary) and anti-Tpl2 M-20 (with AlexaFluor 594 secondary) with a DAPI counterstain. Carets denote Tpl2 co-staining with NP^dim^ cells; arrowheads denote Tpl2 co-staining with NP^bright^ alveolar cells. Data are representative of at least 3 mice. (C) Representative flow plot from lung cells magnetically depleted with biotinylated antibodies against hematopoietic cells (CD45), alveolar macrophages (CD45 and CD16/32), endothelial cells (CD31), erythroid cells (Ter119), club cells (integrin β4), and distal lung progenitor cells (integrin β4) prior to staining for distinction between AECI and AECII. AECI (EpCAM^+^CD45.2^-^Podoplanin^+^) and AECII (EpCAM+CD45.2-) populations were gated as shown and sorted for RT-PCR analyisis of Tpl2. (D) Naïve splenic CD4^+^ and CD8^+^ T cells, bone marrow-derived macrophages (BMDM), adherence purified peritoneal exudate cells (PECs), neutrophils, and lung alveolar epithelial cells (AECI and AECII) were isolated from WT, age- and sex-matched mice as in C. Tpl2 expression was measured by RT-PCR relative to an actin control. All samples were normalized to CD8^+^ T cells, which were arbitrarily designated 1. Data are representative of 3 experiments. (E) Isolated alveolar epithelial cells (AECI and AECII) from WT, age- and sex-matched mice as in C were uninfected or infected with 10^4^ PFU influenza A/x31 for 3 days. Tpl2 expression was quantified by RT-PCR. N = 4. (F) LET1 cells were cultured for 24 hours then left uninfected or infected with a MOI of 0.05, 0.25, 0.5, or 1 of influenza A/x31 (H3N2) for 24 hours. RNA lysates were collected after 24 hours, and Tpl2 expression was measured by RT-PCR. Pooled data from 3 experiments. Error bars represent means ± SEM. Error bars represent means ± SEM. *p < 0.05, ** *p* < 0.01, ***p < 0.001, ****p < 0.0001; one-way ANOVA.

We previously demonstrated that Tpl2 ablation increases morbidity and mortality to influenza A virus infection in mice, and bone marrow chimera experiments suggested that Tpl2 functions within the non-hematopoietic compartment to restrict early virus replication [18]. The aim of this study was to determine if Tpl2 contributes to host defense against influenza infection within alveolar epithelial cells. To confirm Tpl2 expression within epithelial cells, the primary target and replicative niche for influenza A virus [25], WT mice were infected with 10^4^ plaque-forming units (PFU) of mouse-adapted influenza virus A/HK-x31 (H3N2; x31). Lungs were harvested at 3 dpi, and Tpl2 expression and localization within the lung epithelium were assessed by fluorescence microscopy. Tpl2 expression was observed in both type I and type II alveolar epithelial cells (AECI/AECII) (Fig 1A). Influenza A nucleoprotein (NP) staining revealed prominent viral antigens along the bronchioles and dispersed in cells throughout the alveolar compartment. In the alveolar compartment, some cells were densely stained with NP, while others were dimly stained, demonstrating varied viral loads (Fig 1B). Tpl2 co-staining revealed its colocalization with both dimly (Fig 1B, carets) and brightly stained (Fig 1B, arrowheads) alveolar cells.

To quantitate Tpl2 expression in epithelial cells, AECI and AECII were isolated from WT lungs of uninfected and influenza-infected mice, and Tpl2 expression was assessed by real-time PCR [34]. Briefly, a single-cell suspension of lung epithelial cells was obtained after lung digestion with dispase. Isolated lung cells were magnetically depleted using biotinylated antibodies against hematopoietic cells (CD45), alveolar macrophages (CD45 and CD16/32), endothelial cells (CD31), erythroid cells (Ter119), club cells, and distal lung progenitor cells (integrin β4) as previously described [28]. After magnetic depletion of contaminating cell types, the remaining population of cells was stained with CD45.2, EpCAM, and Podoplanin (Pdpn) to distinguish AECIs and AECIIs (Fig 1C). Tpl2 expression trended higher in isolated AECII compared to AECI in uninfected mice (Fig 1D). Both pulmonary epithelial cell types exhibited visibly increased Tpl2 expression by 3 days post influenza infection by immunohistochemistry (Fig 1A, middle and bottom panels), and isolated AECI and AECII cells also showed influenza-induced trending increases in Tpl2 expression that did not reach statistical significance (Fig 1E). Influenza-induced Tpl2 expression in epithelial cells was also examined using the AECI cell line, LET1. Notably, Tpl2 expression was significantly increased in LET1s infected with influenza A/x31 at a MOI of 0.25 and 0.5 (Fig 1F).

### Tpl2 expression within lung epithelial cells is largely dispensable for host resistance to influenza A infection

**Fig 2:**
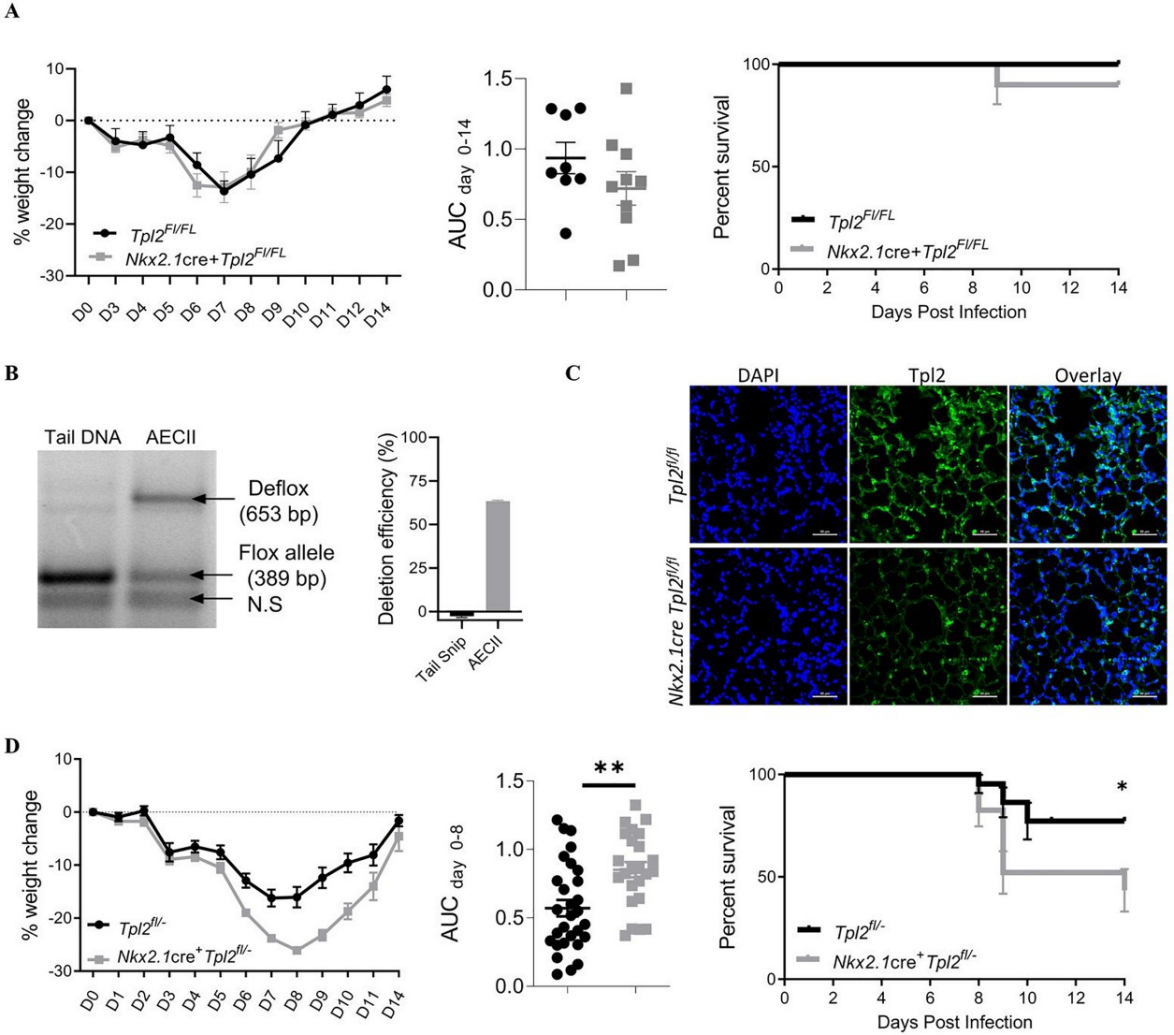
Contribution of Tpl2 expression within pulmonary epithelial cells to morbidity and mortality during influenza infection. (A) *Tpl2*^*fl/fl*^ and *Nkx2*.*1cre*^+^*Tpl2*^*fl/fl*^ conditional knockout mice were infected intranasally with 10^4^ PFU influenza A/x31. Morbidity (left panel) was assessed by daily weight loss. Significance of weight loss was determined by area under the curve (AUC) analysis with an unpaired two-tailed T-test (middle panel). Survival curve (right panel) of the same Tpl2^fl/fl^ and Nkx2.1cre^+^Tpl2^fl/fl^ conditional knockout mice after IAV infection. Mean survival = 100% for *Tpl2*^*fl/fl*^ and 90% for *Nkx2*.*1cre*^+^*Tpl2*^*fl/fl*^; Log-rank (Mantel-Cox) test. Cre^-^ N = 8, Cre^+^ N = 10. Data representative of 2 experiments. Error bars represent means ± SEM. (B) Representative image from 3 mice in which tail snip DNA and DNA from isolated AECII were subjected to genomic amplification of the defloxed allele versus the intact floxed allele. By comparison, *Tpl2* was undeleted in tail DNA where Nkx2.1-cre expression is absent. Quantification of deletion efficiency in *Nkx2*.*1cre*^+^*Tpl2*^*fl/fl*^ mice is shown to the right. N = 3 mice. (C) Immunofluorescence staining of Tpl2 in lungs of *Nkx2*.*1cre*^+^*Tpl2*^*fl/fl*^ and *Tpl2*^*fl/fl*^ mice as in Fig 1A. (D) *Tpl2*^*fl-*^ and *Nkx2*.*1cre*^+^ *Tpl2*^*fl-*^ conditional knockout mice were infected intranasally with 10^4^ PFU influenza A/x31. Morbidity was assessed by daily weight loss (left panel). Significance of weight loss was determined by area under the curve (AUC) analysis with an unpaired two-tailed t-test (middle panel). Cre- N = 29, cre+ N = 24. Pooled data from 3 experiments. Survival curve (Right panel) of *Tpl2*^*fl/-*^ and *Nkx2*.*1cre*^+^ *Tpl2*^*fl/-*^ conditional knockout mice after IAV infection. *p-value = 0.0199. Mean survival = 77.2% for *Tpl2*^*fl/-*^ and 43.3% for *Nkx2*.*1cre*^+^ *Tpl2*^*fl/-*^; Log-rank (Mantel-Cox) test. Cre- N = 22, cre+ N = 23. Pooled data from 3 experiments; Error bars represent means ± SEM. *p < 0.05, **p < 0.005.

To determine if Tpl2 functions in lung epithelial cells to protect against influenza infection, we generated a lung-specific conditional knockout strain with cre expression under the promoter control of *Nkx2*.*1*. Nkx2.1 is a critical transcription factor expressed during early lung development [35, 36]. By utilizing *Nkx2*.*1* as a lung-specific *cre* promoter, we restricted Tpl2 ablation to the epithelial cells of the lower respiratory tract. To test Tpl2 functionality within the lower pulmonary epithelial cells during influenza infection, *Nkx2*.*1*cre^+^*Tpl2*^flox/flox^ mice were infected with 10^4^ PFU influenza A/HK-x31. There was no significant difference in weight loss (Fig 2A, left) or survival (Fig 2A, right) between *Nkx2*.*1cre*^+^*Tpl2*^fl/fl^ mice and *Tpl2*^fl/fl^ control mice.

Due to the absence of a phenotype, we assessed the deletion efficiency of *Tpl2* within isolated AECIIs, which were preferentially analyzed due to the difficulty in isolating AECIs. We determined that AECII from *Nkx2*.*1cre*^+^*Tpl2*^fl/fl^ mice had an average deletion efficiency of ~63% calculated as: (intensity of the defloxed allele / the intensity of the deflox allele + floxed allele) x 100% (Fig 2B). We could not determine deletion efficiency for AECI via this strategy due to low yields of isolated cells. Therefore, we also performed immunofluorescence staining for Tpl2 within these mice and found a similar reduction in Tpl2 expression in AECI cells as defined by morphology (Fig 2C). Specifically, the average corrected total cell fluorescence was 130,481 for *Tpl2*^*fl/fl*^ AECI versus 69,472 for *Nkx2*.*1cre Tpl2*^*fl/fl*^ AECI, as determined by densitometry. This represents a deletion efficiency of approximately 47%. Notably, heterozygous mice missing a single copy of *Tpl2* (50% genetic deletion) can lack a phenotype and are sometimes used as negative controls for *Tpl2*^*-/-*^ mice [20], suggesting that 63% deletion may be insufficient to trigger a *bona fide* phenotype. Anecdotal and recent studies have suggested that crossing a null allele onto a cre-line can improve deletion efficiency [37, 38]; therefore, we crossed *Nkx2*.*1cre*^+^*Tpl2*^fl/fl^ with *Tpl2*^*-/-*^ mice to generate *Nkx2*.*1*cre^+^*Tpl2*^*flox/-*^ mice with littermate controls (hereon, referred to as *Nkx2*.*1*cre^+^*Tpl2*^fl/-^ and *Tpl2*^fl/-^). As a result, the calculated deletion efficiency within AECII cells improved to 85.5%, whereas the calculated deletion efficiency within AECI cells improved to 73.5%.

*Nkx2*.*1*cre^+^*Tpl2*^fl/-^ and *Tpl2*^fl/-^ mice were infected with 10^4^ PFU influenza A/HK-x31 (x31), and weights were monitored over 14 days. *Nkx2*.*1*cre^+^*Tpl2*^fl/-^ mice exhibited significantly more weight loss than control *Tpl2*^fl/-^ mice, with morbidity peaking at 8 dpi (Fig 2D, left). In a fraction of the mice in both strains, weight loss was accompanied by labored breathing (dyspnea), which necessitated euthanasia according to humane endpoints of the study. Notably, *Nkx2*.*1*cre^+^*Tpl2*^fl/-^ mice also displayed higher mortality when compared to *Tpl2*^fl/-^ mice (Fig 2D, right).

**Fig 3:**
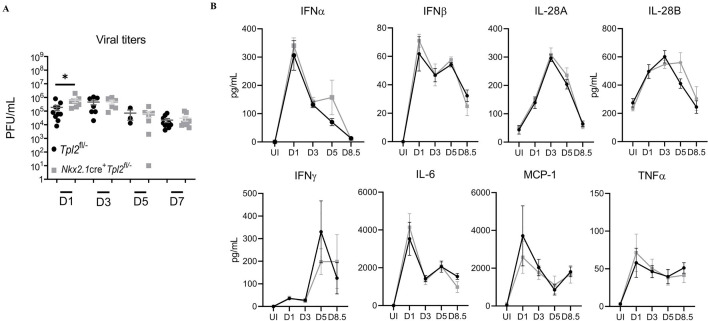
*Nkx2*.*1*cre^+^*Tpl2*^fl/-^ mice have impaired early viral control but normal inflammatory cytokine profiles. (A) Lung viral titers determined by plaque assay in mice infected with 10^4^ pfu influenza A/x31 for the indicated times. Pooled data from 4 separate experiments, 1 experiment per time point. Mice per group- D1: *Tpl2*^fl/-^, N = 10. *Nkx2*.*1*cre^+^*Tpl2*^fl/-^, N = 8; D3: *Tpl2*^fl/-^, N = 7. *Nkx2*.*1*cre^+^*Tpl2*^fl/-^, N = 6; D5: *Tpl2*^fl/-^, N = 3, *Nkx2*.*1*cre^+^*Tpl2*^fl/-^, N = 7; D7: *Tpl2*^fl/-^, N = 10, *Nkx2*.*1*cre^+^*Tpl2*^fl/-^, N = 9. (B) Cytokine expression in mice treated as in **A** was determined by Procartaplex immunoassay; data representative of 2 experiments. Mice per group—UI *Tpl2*^fl/-^, N = 4, *Nkx2*.*1*cre^+^*Tpl2*^fl/-^, N = 4; D1: *Tpl2*^fl/-^, N = 3, *Nkx2*.*1*cre^+^*Tpl2*^fl/-^, N = 3; D3: *Tpl2*^fl/-^, N = 6, *Nkx2*.*1*cre^+^*Tpl2*^fl/-^, N = 6; D5 *Tpl2*^fl/-^, N = 3, *Nkx2*.*1*cre^+^*Tpl2*^fl/-^, N = 3; D8.5: *Tpl2*^fl/-^, N = 4, *Nkx2*.*1*cre^+^*Tpl2*^fl/-^, N = 5; Error bars represent means ± SEM. *p < 0.05, **p < 0.01, ***p < 0.001, ****p < 0.0001; one-way ANOVA unless otherwise noted.

To determine the cause of increased morbidity and mortality in influenza-infected *Nkx2*.*1*cre^+^*Tpl2*^fl/-^ mice, we harvested lung homogenates at 1, 3, 5, and 7 dpi from influenza-infected *Nkx2*.*1*cre^+^*Tpl2*^fl/-^ and *Tpl2*^fl/-^ control mice. We noted a modest, statistically significant increase in viral titers at 1 dpi in the *Nkx2*.*1*cre^+^*Tpl2*^fl/-^ mice, but the increase was resolved by 3 dpi and remained comparable to the *Tpl2*^fl/-^ mice through the study’s end (Fig 3A). Therefore, increased morbidity and mortality in *Nkx2*.*1*cre^+^*Tpl2*^fl/-^ mice could not be attributed to an inability to control viral replication. Some influenza infections cause mortality due to an overwhelming immune response rather than uncontrolled viral titers [39]. To address this possibility, we measured the abundance of inflammatory mediators in lung homogenates over a similar time course. To our surprise, no significant differences in cytokine production were observed in *Nkx2*.*1*cre^+^*Tpl2*^fl/-^ mice (Fig 3B).

**Fig 4:**
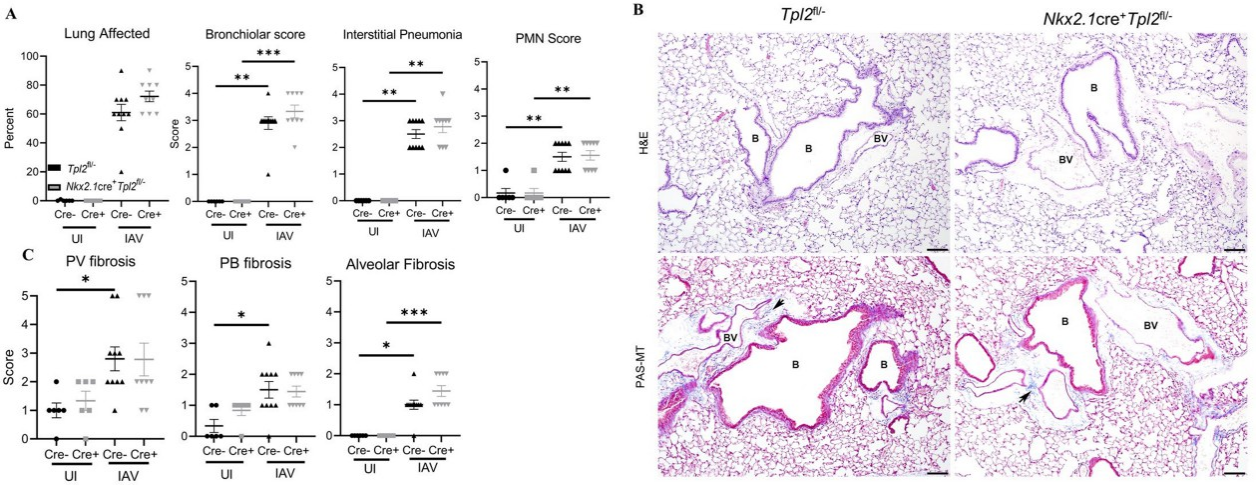
*Nkx2*.*1*cre^+^*Tpl2*^fl/-^ mice have normal pulmonary histological profiles at 7 dpi with influenza A virus. (A) Inflammation scoring of *Tpl2*^*fl/-*^ and *Nkx2*.*1cre*^+^*Tpl2*^*fl/-*^ mice at 8.5 dpi with 10^4^ PFU influenza A/x31. Lungs sections were stained with hematoxylin and eosin and scored by a pathologist blinded to sample identity. *Tpl2*^*fl/-*^, N = 16 (6 controls, 10 experimental). *Nkx2*.*1cre*^+^*Tpl2*^*fl/-*^, N = 15 (6 controls, 9 experimental). (B) Representative images of *Tpl2*^*fl/-*^ and *Nkx2*.*1cre*^+^*Tpl2*^*fl/-*^ lung sections stained with hematoxylin and eosin (H&E) at 8.5 dpi (top panels) and Periodic Acid Schiff, counterstained with hematoxylin and Masson’s trichrome stain (PAS-MT) at 8.5 dpi (bottom panels). B (Bronchiole), BV (Blood Vessel). Arrows point to areas of fibrosis as indicated by blue staining in PAS-MT panels. Data representative of 2 experiments. (C) Fibrosis scoring. Lung sections as in B were scored for fibrosis by a pathologist blinded to sample identity. Scoring was on a scale of 1–5 of the worst affected structure relative to normal thickness. Perivascular (PV), Peribronchiolar (PB), Alveolar fibrosis. *Tpl2*^*fl/-*^, N = 16 (6 controls, 10 experimental). *Nkx2*.*1cre*^+^*Tpl2*^*fl/-*^, N = 15 (6 controls, 9 experimental). Data representative of 2 experiments. Error bars represent means ± SEM. *p < 0.05, ** *p*< 0.01, ***p < 0.001; Kruskal-Wallis nonparametric test with Dunn’s multiple comparisons post-test.

We next assessed pulmonary changes associated with increased morbidity and mortality in *Nkx2*.*1*cre^+^*Tpl2*^fl/-^ mice. Lung samples harvested at 8.5 dpi from *Nkx2*.*1*cre^+^*Tpl2*^fl/-^ and *Tpl2*^fl/-^ littermate controls were submitted for histopathology to characterize infection-induced pathologic changes in the bronchioles, alveoli, and pleura. Influenza infection led to higher scores for all inflammation metrics, including percent lung affected, bronchiolar, interstitial pneumonia, and PMN scores, although no significant differences in influenza-induced inflammation were noted between genotypes (Fig 4A and 4B).

Multiple lines of evidence have recently implicated Tpl2 in restraining fibrotic responses, both in intestinal myofibroblast-mediated fibrosis in the gut [23] and in bleomycin-induce lung fibrosis [22]. Because pulmonary fibrosis can impair lung function during influenza infection [40], we assessed whether Tpl2 protects against influenza-induced lung injury by inhibiting pulmonary fibrosis. However, no significant differences in pulmonary fibrosis were noted in the major regions of the lung (Fig 4B and 4C). Consistently, lung collagen levels, as determined by hydroxyproline assay at 8 dpi, showed no significant difference between strains (S1A Fig), nor were there any differences in key lung fibrotic markers, *Ptges2*, *Reg3g*, and *Timp1* at 8.5 dpi (S1B Fig). To determine if *Tpl2* ablation in non-hematopoietic cells indirectly caused systemic hematologic or metabolic effects that could be responsible for increased morbidity and mortality, we examined several clinical markers of systemic disease at the peak of morbidity (8.5 dpi) in one large representative cohort of mice. The circulating number and frequency of immune cells was not different compared to *Tpl2*^fl/-^ mice (S2 Fig). Additionally, there was no indication that anemia was a factor leading to the increased morbidity and mortality in *Nkx2*.*1*cre^+^*Tpl2*^fl/-^ mice (S2B Fig). Collectively, *in vivo* studies suggest that, despite influenza-induced expression of Tpl2 in lung epithelial cells, epithelial cell-intrinsic Tpl2 appears to only modestly contribute to host protection against influenza infection. Given that *Tpl2* ablation has been linked to overexpression of type I IFNs [17] and reduced expression of type III IFNs [18], we next examined the Tpl2-dependent regulation of IFN production in virus-infected lung epithelial cells *in vitro*.

### Tpl2 kinase activity does not regulate type I IFN responses in the AECI cell line, LET1, in response to influenza infection

**Fig 5:**
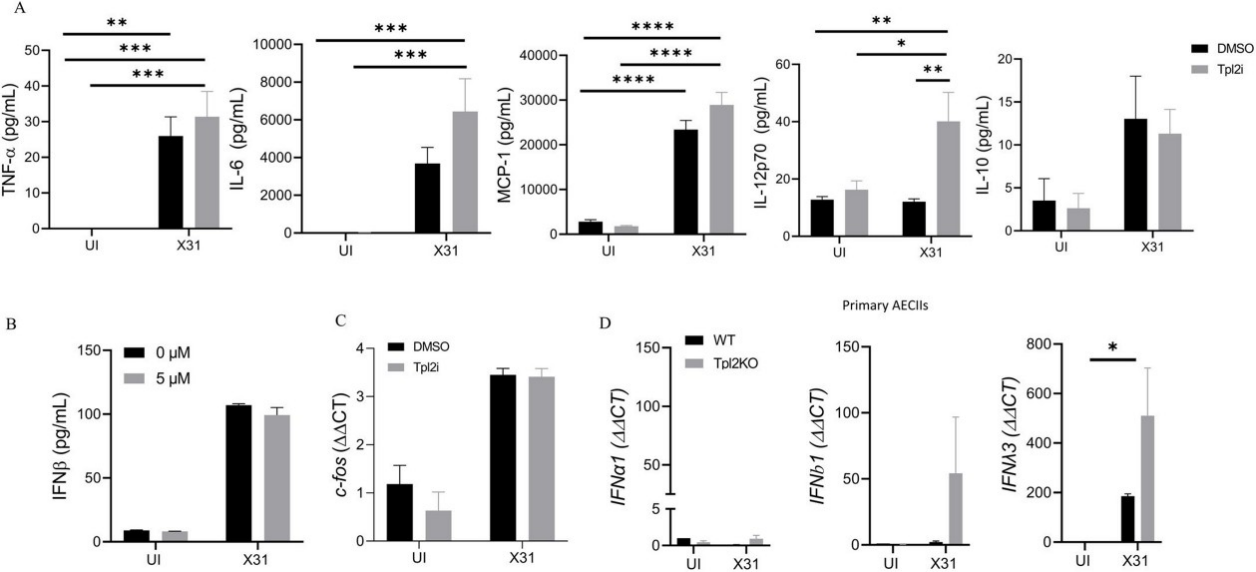
Tpl2 does not regulate type I IFN responses in LET1s in response to influenza infection. LET1s were cultured for 24 hours prior to stimulation with a MOI of 0.5 of influenza A/x31 (H3N2) for 24 hours after a 30-minute pre-treatment with DMSO vehicle control or Tpl2 inhibitor (5 μM). (A) Cytokine expression measured by CBA analysis from supernatants collected 24 hpi. Pooled data from 3 experiments. (B) IFN-β secretion was measured by ELISA from supernatants collected 24 hpi. Data are representative of 3 independent experiments. (C) c-fos levels were quantified by RT-PCR from RNA lysates collected 24 hpi. Pooled data from 3 experiments. (D) Primary AECIIs were isolated from influenza-infected WT or *Tpl2*^*-/-*^ mice at 3 dpi. IFN-α1, IFN-β1, and IFN-λ3 expression levels were quantified by RT-PCR from primary AECII RNA lysates.

Previous studies have shown that Tpl2 is required for the induction of IFNs in pDCs [17, 18] but that Tpl2 negatively regulates type I IFNs in macrophages and pDCs in response to TLR stimulation [17]. To our surprise, *Nkx2*.*1*cre^+^*Tpl2*^fl/-^ mice produced normal levels of IFNs in response to IAV infection (Fig 3B). To gain insight into the Tpl2-dependent regulation of IFNs in pulmonary epithelial cells, we took advantage of the AECI cell line, LET1. AECI cells play a vital role in influenza restriction without the influence of the hematopoietic compartment [31]; however, they are notoriously difficult to isolate and study *in vitro*. The LET1 cell line shows similar antiviral responses to primary AECI [31] and was used to explore the function of Tpl2 in alveolar epithelial cells during influenza infection. To define Tpl2-dependent regulation of inflammatory responses within AECI, we infected LET1s with 0.5 MOI of influenza A/x31 for 24 hr in the presence or absence of a selective Tpl2 inhibitor. We found that TNF, IL-6, and MCP-1 were not significantly different in Tpl2-inhibited influenza-infected cells compared to vehicle-treated influenza-infected controls (Fig 5A). A significant increase in IL-12 secretion was noted in Tpl2-inhibited and influenza-infected LET1s (Fig 5A), consistent with previous studies showing a negative regulatory role for Tpl2 in IL-12 expression by bone marrow-derived macrophages (BMDM) and bone marrow-derived dendritic cells (BMDC) [41]. However, in Tpl2-inhibited influenza infected cells there was no difference in the production of type I IFNs as measured by IFN-β compared to uninhibited controls (Fig 5B). IL-28B (IFN-λ3) was not consistently detected at the protein level by ELISA in the same culture supernatants.

The negative regulation of IL-12 by Tpl2 can occur via IL-10-dependent or—independent mechanisms in macrophages and dendritic cells [41]. However, despite increased IL-12 upon Tpl2 inhibition (Fig 5A), there was no significant difference in IL-10 (Fig 5A), suggesting that Tpl2 regulates IL-12 via an IL-10-independent mechanism in LET1s. In macrophages, negative regulation of IL-12 relies upon Tpl2-dependent *c-fos* changes [41]. *c-fos* is induced by Tpl2 in macrophages and, in turn, negatively regulates IL-12 production in the presence or absence of IL-10 [41]. Therefore, we determined whether Tpl2-dependent *c-fos* also regulated IL-12 production in type I epithelial cells. Equivalent *c-fos* expression in LET1s treated with Tpl2 inhibitor or vehicle control (Fig 5C) demonstrated that the Tpl2-dependent negative regulation of IL-12 in LET1s occurs independently of both IL-10 and *c-fos*.

To determine whether Tpl2 regulates the antiviral immune response of AECII, we were able to isolate primary AECII with sufficiently high yield from WT and *Tpl2*^*-/-*^ mice at 3 dpi with influenza A/x31 to allow for transcriptional analysis (Fig 1D and 1E). We found that *Tpl2*-deficient primary AECII isolated from influenza-infected mice expressed similar levels of both type I and type III IFNs compared to WT (Fig 5D). Overall, these data suggest that Tpl2 is dispensable for the production of antiviral IFNs in both AECI and AECII upon influenza virus sensing.

## Discussion

We found that pulmonary epithelial cells within the lower respiratory tract (AECIs and AECIIs) increased Tpl2 expression upon influenza infection (Fig 1A). These data are consistent with previous studies showing that Tpl2 is transcriptionally induced and activated upon influenza infection in the human embryonic kidney cell line, 293T [24]. Additionally, previous studies from our own and other groups have shown that Tpl2 is an IFN-induced gene in response to viral infection [42, 43].

Tpl2 expression is critical for an effective host anti-viral response, as global ablation of Tpl2 leads to increased morbidity and mortality to an otherwise low pathogenicity strain of influenza [18, 44]. Notably, morbidity was associated with an overactive immune response rather than impaired anti-viral control [44]. In addition, increased morbidity correlated with increased expression of IFN-β, which was also positively correlated with CCL2, CXCL1, and the expression of inducible nitric oxide synthase (iNOS, encoded by *Nos2*) [44]. This same study also indicated that Tpl2 ablation within radioresistant cells was required for the initial upregulation of proinflammatory cytokines upon influenza infection. Therefore, the current study specifically addressed the contribution of Tpl2 expressed within radioresistant pulmonary epithelial cells to influenza-induced cytokine dysregulation.

Tpl2 conditional ablation with the *Nkx2*.*1* promoter was used to assess Tpl2 function in epithelial cells. *Nkx2*.*1*cre^+^*Tpl2*^fl/fl^ showed normal weight loss and mortality curves to influenza infection, suggesting that Tpl2 is dispensable for antiviral functions in epithelial cells. However, because Tpl2 deletion efficiency in that model was only ~47% in AECI and 63% and AECII, we questioned whether we would be able to detect a phenotype if one existed. Therefore, we intercrossed those mice with *Tpl2*^*-/-*^ mice to preemptively delete Tpl2 on one allele to improve deletion efficiency within the epithelial compartment and to also query the phenotype of mice heterozygous for Tpl2. In this line of *Nkx2*.*1*cre^+^*Tpl2*^fl/-^ mice we did observe a modest increase in morbidity and mortality compared to *Tpl2*^fl/-^ control mice, suggesting a minor contribution of epithelial cell-expressed Tpl2 to host protection against influenza. However, we were not able to localize a biological mechanism responsible for the difference in morbidity and mortality, despite analysis of various parameters, including inflammatory cytokine production, pathology, immune cell infiltration, fibrosis markers and anemia. A modest, but statistically significant increase in viral titers was observed at 1 dpi which normalized by 3 dpi and was therefore unable to explain morbidity and mortality later at 9 dpi. Therefore, the mechanism for the modest differences observed are currently unknown.

It is noteworthy that *Tpl2*^*fl/-*^ mice showed approximately 23% mortality in response to infection with 10^4^ pfu x31 infection, which is unusual considering that mortality has not previously been observed at this dose [18], even using the same influenza stock. This suggests that there is a gene dosage effect of Tpl2 ablation in response to influenza infection, such that heterozygote mice with 50% reduction in Tpl2 display increased mortality. This further suggests that the deletion efficiency of~47–63% in epithelial cells of *Nkx2*.*1*cre^+^*Tpl2*^fl/fl^ mice should be sufficient to capture a Tpl2-dependent phenotype in epithelial cells if one existed. Therefore, we conclude from this data that Tpl2 is dispensable in epithelial cells for host protection to influenza infection.

During our investigation of Tpl2’s contribution in purified, influenza-infected epithelial cells utilizing LET1s as a model, we detected Tpl2-dependent inhibition of IL-12p70 production but not IFN-β (Fig 5). Notably, IFN-λ expression was not observed consistently at the protein level in influenza-infected LET1s. The negative regulation of IL-12 by Tpl2 is consistent with its negative regulation in *Tpl2*^*-/-*^ dendritic cells in response to TLR stimulation [41]. In *Tpl2*^*-/-*^ BMDMs and BMDCs, impaired Tpl2-dependent ERK induction of *c-fos* permits increases in IL-12 [41]. Therefore, we hypothesized that Tpl2-dependent *c-fos* expression also negatively regulates IL-12 during influenza infection by the same mechanism. However, *c-fos* levels were unchanged in Tpl2 inhibitor-treated LET1s, arguing that Tpl2 inhibits IL-12 independently of *c-fos*. Thus, the increase in IL-12 within LET1s appears to be regulated by a mechanism distinct from that observed in BMDMs and BMDCs.

This model of epithelial cell-specific ablation failed to recapitulate the severe influenza-induced disease seen in mice with global Tpl2 ablation, demonstrating that Tpl2 activity in pulmonary epithelial cells is dispensable for the major antiviral responses to influenza infection *in vitro* and *in vivo*. Therefore, the question arises as to ‘what Tpl2-expressing cell type(s) play a dominant role in suppressing influenza exacerbations in mice?’ It is well known that a variety of cell types can be directly infected by influenza A virus and/or produce interferons in response to influenza virus sensing. For example, plasmacytoid dendritic cells (pDCs), have been shown to produce type I and type III interferons in response to influenza infection [45]. In fact, Jewell *et al*. showed that pDCs contribute significantly to the overall type I IFN response during influenza infection, since pDC depletion reduced influenza-induced type I IFNs by greater than two-fold [46]. Hematopoietic cells such as natural killer cells, neutrophils, and some subsets of human and mouse conventional dendritic cells that have known Tpl2-dependent functions in interferon production and/or restriction of viral replication are recruited in response to viral infection and may also play an important role. However, one major contributing cell type may be alveolar macrophages. During homeostasis, alveolar macrophages adhere to type I and type II alveolar epithelial cells; during influenza infection alveolar macrophages upregulate interferons and are critical for protecting the epithelial barriers [47]. In fact, alveolar macrophages are major producers of type I IFNs during and after influenza infection [48, 49]. Therefore, the role of Tpl2 in alveolar macrophages will be investigated in future studies. Given the cell-type and stimulus-specific nature of Tpl2 [9], other radioresistant cell types, such as stromal cell populations, including endothelial cells and myofibroblasts also need to be considered. Previous studies have shown important roles for Tpl2 in intestinal [23] and lung myofibroblast [22] and lung endothelial cells [50]. However, whether and how Tpl2 functions in these cell types to induce protective antiviral responses is currently unknown.

## Supporting information

S1 Fig Lung-specific Tpl2 ablation does not alter pulmonary fibrosis markers. (A) Collagen levels in *Nkx2*.*1*cre^+^*Tpl2*^*flox/-*^ and control mice at 8 dpi with 10^4^ PFU influenza A/x31 measured by hydroxyproline production within the lung. N = 1 experiment. (B) *Ptges2*, *Reg3g*, *Timp1* expression levels were quantified by RT-PCR from RNA lysates collected from uninfected and D8.5 lung homogenates. Unpaired two-tailed T-test. Data are representative of 2 experiments.
(TIF)

S2 Fig Lung-specific *Tpl2* ablation does not induce hematological or metabolic changes. (A) Complete blood count (CBC) from *Tpl2*^*fl-*^ and *Nkx2*.*1*cre^+^*Tpl2*^*fl/-*^ mice at 8.5 dpi with 10^4^ PFU influenza A/x31 measuring immune cell volume and frequency collected by terminal cardiac puncture N = 1. (D) Anemia-associated markers analyzed by CBC. RDW- Red blood cell distribution width, Hct- hematocrit, Hgb-hemoglobin, MCHC- Mean corpuscular hemoglobin concentration, MCH- mean corpuscular hemoglobin, MCV- Mean corpuscular volume, MPV- Mean platelet volume. N = 5 mice per group.
(TIF)
