# Supplementary figures and images for: Influenza-induced Tpl2 expression within alveolar epithelial cells is dispensable for host viral control and anti-viral immunity
Source: PLoS One. 2022 Jan 20;17(1):e0262832. doi: 10.1371/journal.pone.0262832 (PMC8775564; doi:10.1371/journal.pone.0262832)

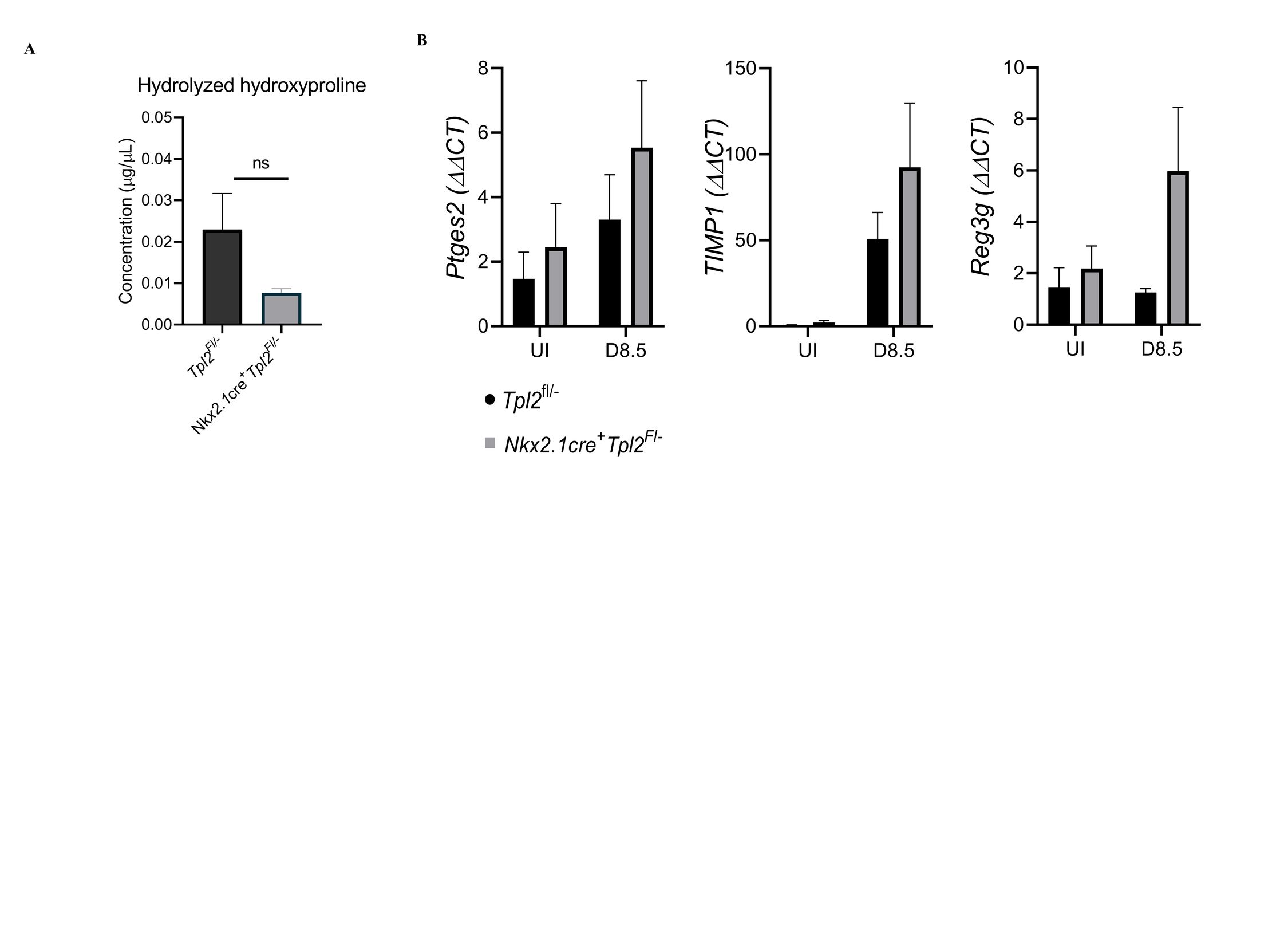

Supplement: S1 Fig — (A) Collagen levels in Nkx2.1cre+Tpl2flox/- and control mice at 8 dpi with 104 PFU influenza A/x31 measured by hydroxyproline production within the lung. N = 1 experiment. (B) Ptges2, Reg3g, Timp1 expression levels were quantified by RT-PCR from RNA lysates collected from uninfected and D8.5 lung homogenates. Unpaired two-tailed T-test. Data are representative of 2 experiments. (TIF) [file pone.0262832.s001.tif]

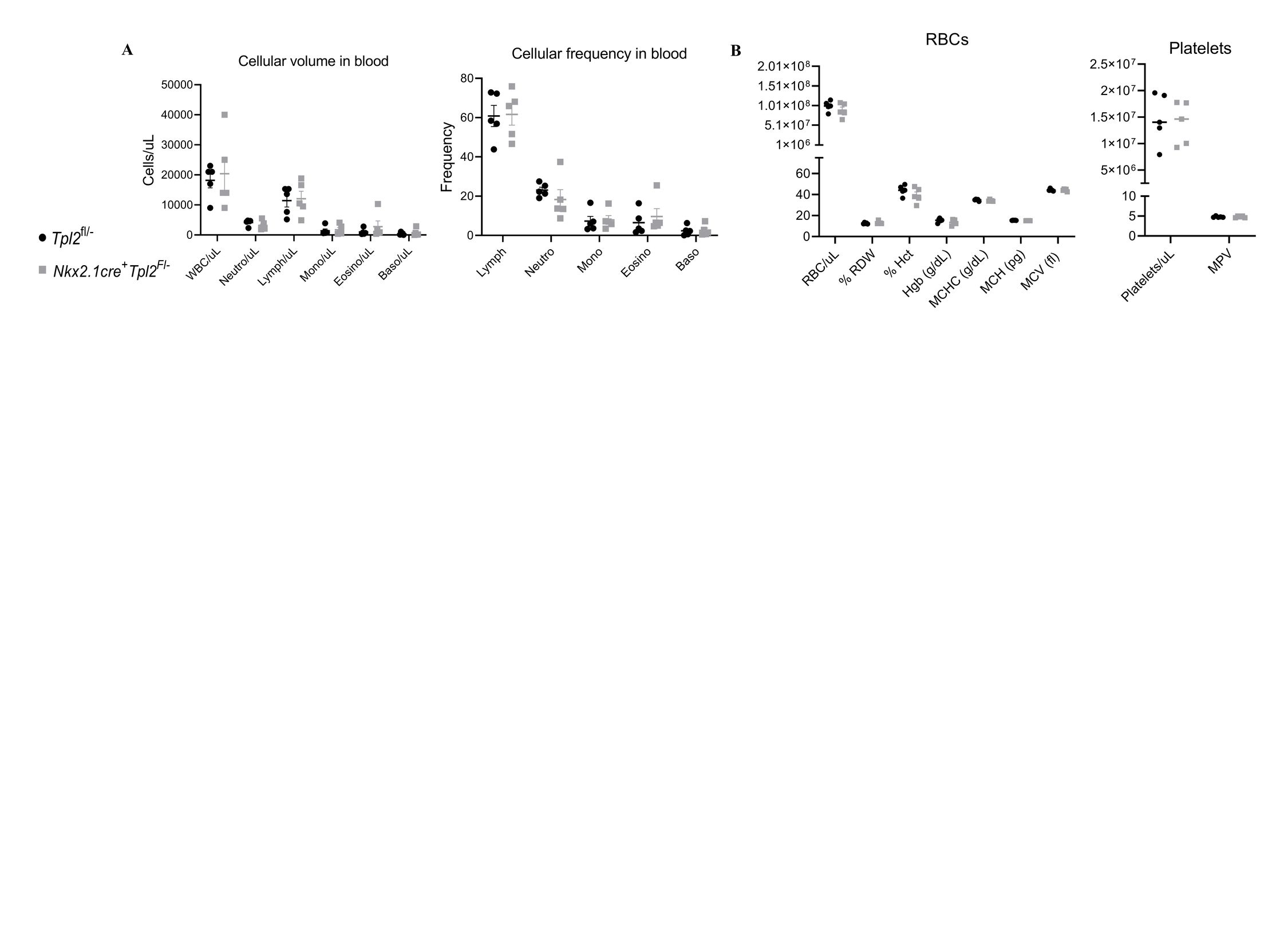

Supplement: S2 Fig — (A) Complete blood count (CBC) from Tpl2fl- and Nkx2.1cre+Tpl2fl/- mice at 8.5 dpi with 104 PFU influenza A/x31 measuring immune cell volume and frequency collected by terminal cardiac puncture N = 1. (D) Anemia-associated markers analyzed by CBC. RDW- Red blood cell distribution width, Hct- hematocrit, Hgb-hemoglobin, MCHC- Mean corpuscular hemoglobin concentration, MCH- mean corpuscular hemoglobin, MCV- Mean corpuscular volume, MPV- Mean platelet volume. N = 5 mice per group. (TIF) [file pone.0262832.s002.tif]
